# Comparison of early complications between the use of a cannulated screw locking plate and multiple cancellous screws in the treatment of displaced intracapsular hip fractures in young adults: a randomized controlled clinical trial

**DOI:** 10.1186/s13018-018-0901-3

**Published:** 2018-08-13

**Authors:** Zhiqiang Wang, Yi Yin, Qingshan Li, Guanjun Sun, Xu Peng, Hua Yin, Yongjie Ye

**Affiliations:** Department of Orthopaedics Surgery, Suining Central Hospital, Suining, 629000 Sichuan China

**Keywords:** Intracapsular hip fracture, Shortening, Complication

## Abstract

**Background:**

The incidence of early postoperative complications of displaced intracapsular hip fractures is high. The purpose of this study was to compare the early postoperative complications and assess the incidence of femoral neck shortening on using a newly designed proximal femoral cannulated screw locking plate (CSLP) versus multiple cancellous screws (MCS) in the treatment of displaced intracapsular hip fractures in young adults.

**Methods:**

Sixty-eight young adult patients with displaced intracapsular hip fractures were randomly assigned to either the CSLP group or the MCS group and treated routinely by internal fixation with either the CSLP or the MCS. Harris Hip Score, nonunion, failure of fixation, overall complications, and femoral neck shortening were recorded and compared.

**Results:**

Two patients (5.88%) in the CSLP group and eight (23.53%) in the MCS group had postoperative nonunion (*P* < 0.05). There was one case (2.94%) of fixation failure in the CSLP group and three cases (8.82%) in the MCS group (*P* > 0.05). Three patients (8.82%) in the CSLP group and 11 (32.35%) in the MCS group had overall complications (*P* < 0.05). Mean femoral neck shortening was 5.10 mm in the vertical plane and 5.11 mm in the horizontal plane in the CSLP group and 11.14 mm in the vertical plane and 10.51 mm in the horizontal plane in the MCS group. Severe femoral neck shortening (≥ 10 mm) did not occur in either the vertical or the horizontal plane in any patient of the CSLP group but occurred in 10 patients (28.57%) in the vertical plane and in 8 (22.86%) patients in the horizontal plane in the MCS group.

**Conclusions:**

Compared with MCS, the use of CSLP in the treatment of displaced intracapsular hip fractures in young adults can reduce the rates of postoperative nonunion and overall complications and minimize femoral neck shortening.

**Trial registration:**

ChiCTR1800016032. Registered 8 May 2018. Retrospectively registered.

## Background

Displaced intracapsular hip fractures in young adults are generally caused by high-energy trauma. Along with a severely damaged blood supply, there is a high risk of postoperative nonunion and femoral head avascular necrosis [1–3]. Various options exist for internal fixation of the hip, including a sliding hip screw/side plate device and multiple cannulated parallel lag screws [4]. However, even with the use of these treatment methods, the incidence of early postoperative complications such as nonunion and failure of fixation is high [5–7].

The use of multiple cancellous screws (MCS) can lead to dynamic compression at the fracture site during axial loading, resulting in a shortened femoral neck. A shortened femoral neck or an offset can cause abductor muscle weakness as a result of a decreased lever arm as well as overall limb shortening, which has been shown to be associated with significantly lower Physical Functioning and Role Physical SF-36 subscores [8–10]. The use of MCS has also been shown to correlate with a decreased quality of life [8], which may be not acceptable in active young adults.

A recent study [11] has shown that compared to three other kinds of internal fixation devices, cannulated screws, DHS, and dynamic condylar screw (DCS), fixed-angle proximal femoral locking plate (PFLP) has the best biomechanical properties, with the highest axial stiffness. However, there are few successful reports focusing on this method in the treatment of displaced femoral neck fractures in young adults.

**Fig. 1:**
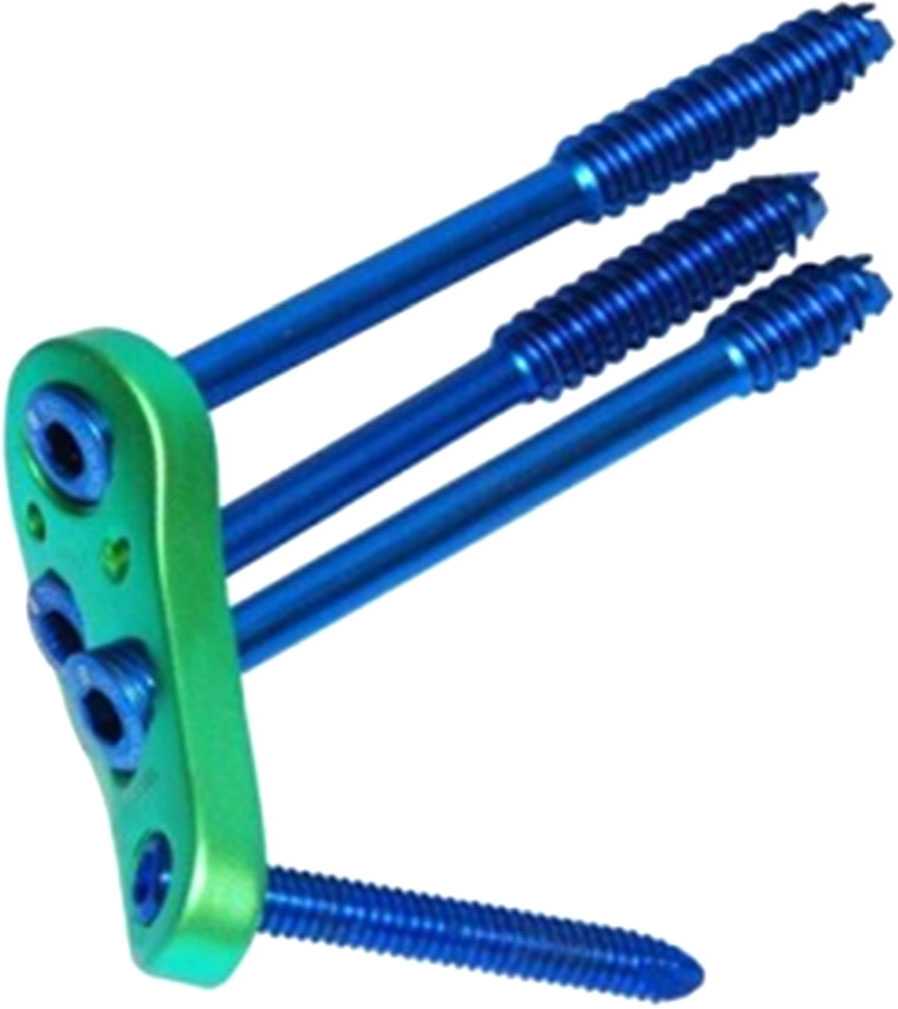
The newly designed cannulated screw locking plate used in this study

Since February 2009, we have been using a newly designed fixed-angle device—a cannulated screw locking plate (CSLP, Xiamen Double Engine Medical Material Co. Ltd)—in the treatment of displaced intracapsular hip fractures in young adults (Garden type III–IV, OTA 31-B2.3 or 31-B3) (Fig. 1). We conducted a randomized controlled study in order to compare postoperative complications and femoral neck shortening on use of the CSLP versus MCS. Historical literature dealing with this question is sparse and this is to our knowledge the first attempt at comparing femoral neck shortening after use of a fixed-angle device versus the conventional fixation method.

We hypothesized that the use of the CSLP in the treatment of displaced intracapsular hip fractures in young adults could reduce the rates of early postoperative nonunion and failure of fixation. In addition, we presumed that the use of the CSLP would minimize postoperative shortening.

## Methods

**Fig. 2:**
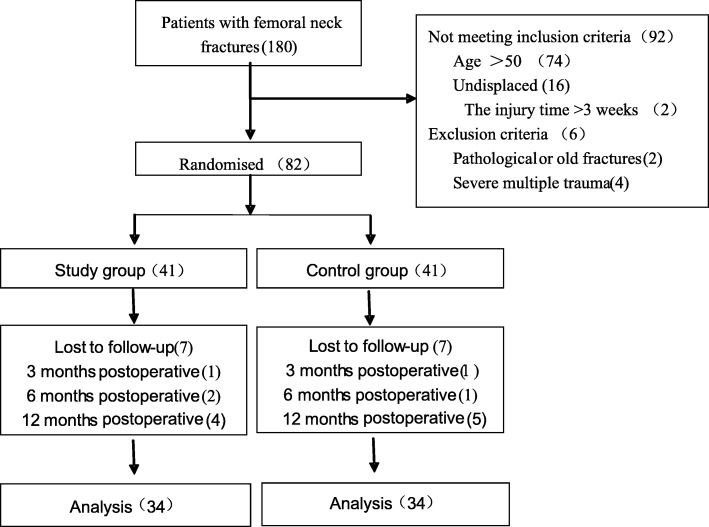
A flow diagram of the patient randomization, follow-up, and subsequent analysis performed in this study

Approval for the study was obtained from our Institutional Review Board. Each patient provided written informed consent, agreeing to participate in this study. A total of 180 patients with intracapsular hip fractures who were hospitalized in our department between February 2009 and December 2017 were enrolled in the study (Fig. 2). The inclusion criteria were (1) age between 18 and 50 years, (2) intracapsular hip fractures resulting from high-energy injuries (e.g., traffic accidents, falls, and sports injuries), (3) recent intracapsular hip fractures (less than < 48 h), (4) displaced intracapsular hip fractures (Garden type III–IV, OTA 31-B2.3 or 31-B3), and (5) surgical approaches of closed or open reduction and internal fixation. The exclusion criteria were (1) pathological or old fractures (injury time > 3 weeks) or undisplaced intracapsular hip fractures (Garden type I–II), (2) severe blood and immune system diseases, (3) severe multiple traumas or a previous history of ipsilateral hip or femur surgery, (4) conditions such as osteoarthritis and post-dysplastic deformities, and (5) follow-up time of less than 1 year. Patients with osteonecrosis, secondary displacement (nonunion), or reoperation were included in the study even if their follow-up time was less than 1 year. Eighty-two patients were randomly assigned to either the CSLP group or the MCS group. Randomization was performed using identical, sealed, opaque envelopes. Patients were treated routinely by internal fixation with either the CSLP or MCS. In both patient groups, surgeries were performed by two experienced orthopedic surgeons (Y.J.Y., Y.Y.). The postoperative Harris Hip Score, postoperative complications, and femoral neck shortening were compared between the two groups.

Patients in both groups were given either general or regional anesthesia while in a supine position on the fracture table with the affected hip elevated by an angle of 10–15°. The traction bed was used for closed reduction, and anteroposterior and lateral radiographs of the femoral neck were obtained using the C-arm X-ray machine. Garden’s alignment index was adopted to evaluate the effectiveness of closed reduction. Reduction was considered acceptable if the angle between the femoral medial cortex and the central axis of the compression trabeculae in medial femoral head was 160–180° on the anteroposterior radiograph and 180° on the lateral radiograph and if there was a maximum difference of 20° in Garden’s alignment index on the lateral radiograph before and after reduction. Otherwise, the effectiveness of the closed reduction was not considered ideal, and internal fixation was considered essential after open reduction. A Watson-Jones approach [12] was used, and the fracture was opened by performing an inverted T-shaped incision in the capsule. A Kirschner wire was used to manipulate the head of the femur.

**Fig. 3:**
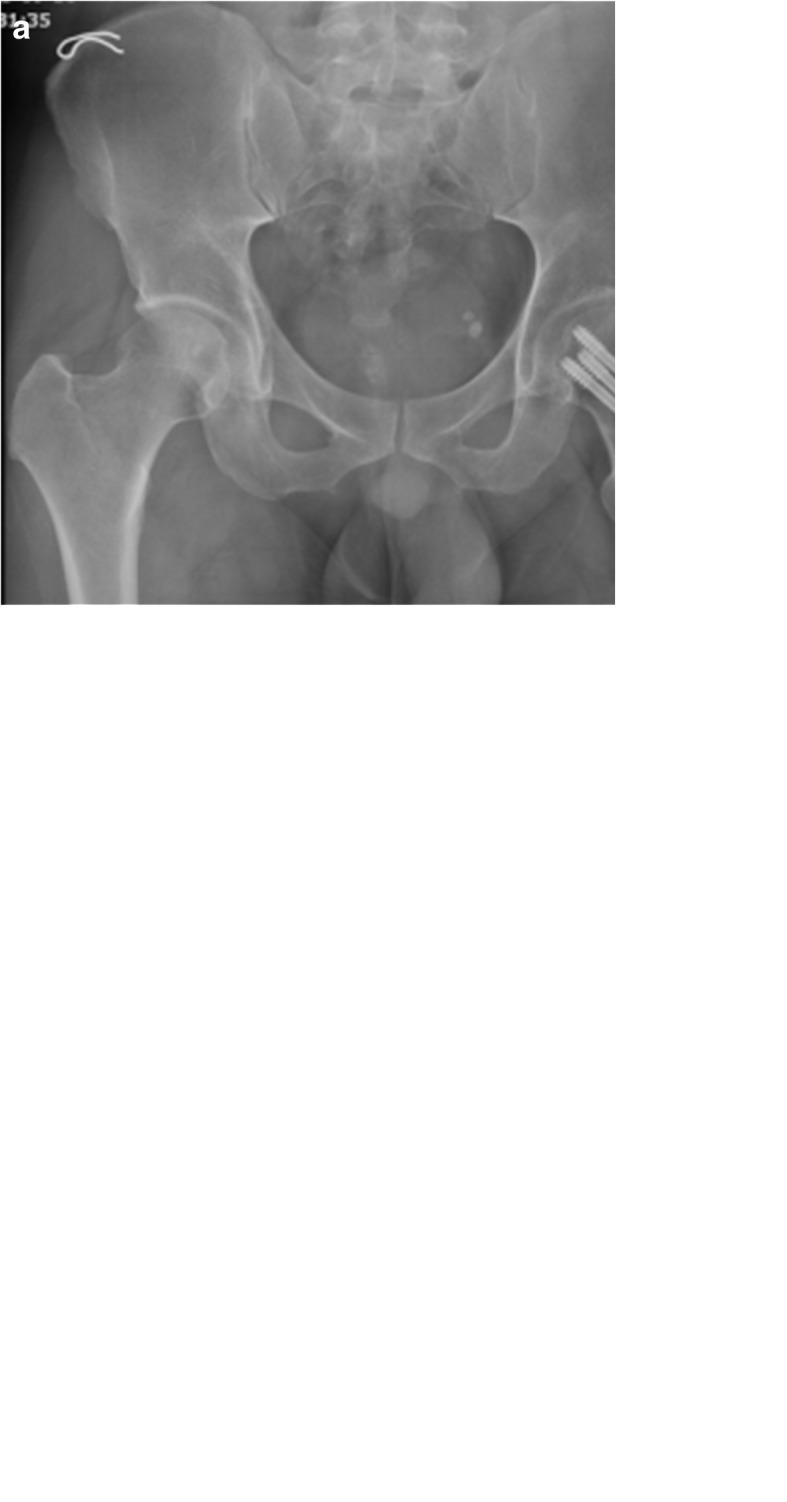
Anteroposterior view of pelvic (**a**) and lateral radiograph of the femoral neck (**b**) after closed reduction and internal fixation with the cannulated screw locking plate. Anteroposterior view of the hip after internal fixation was removed (**c**)

For the patients in the CSLP group, a 5–8-cm downward longitudinal incision was made from the greater trochanter of the femur. The iliotibial band and the vastus lateralis muscle were incised and 4–5 cm of bone surface under the greater trochanter was exposed. Next, the guide wire was drilled into the inferior portion of the femoral neck, and the position of the guide wire in the femoral neck was confirmed under fluoroscopy. The Kirschner wire was placed on a CSLP, and two other guide wires were drilled into the femoral neck. The hollow drill was placed into the drill hole of the guide wire. Locking cannulated screws of appropriate length were selected and tightened. The stabilizing screw was finally secured at the bottom of the locking plate. During the operation, the split gaskets were initially fixed onto the locking holes, and the screws were tightened to enable compression of the fracture ends. The gaskets were removed to lock the screws before a stabilizing screw was finally placed into the distal end of the steel plate. Theoretically, the three screws should be arranged in an inverted triangle in the femoral neck and the average distance from the screw tip to the femur head apex should be 5–10 mm (Fig. 3).

**Fig. 4:**
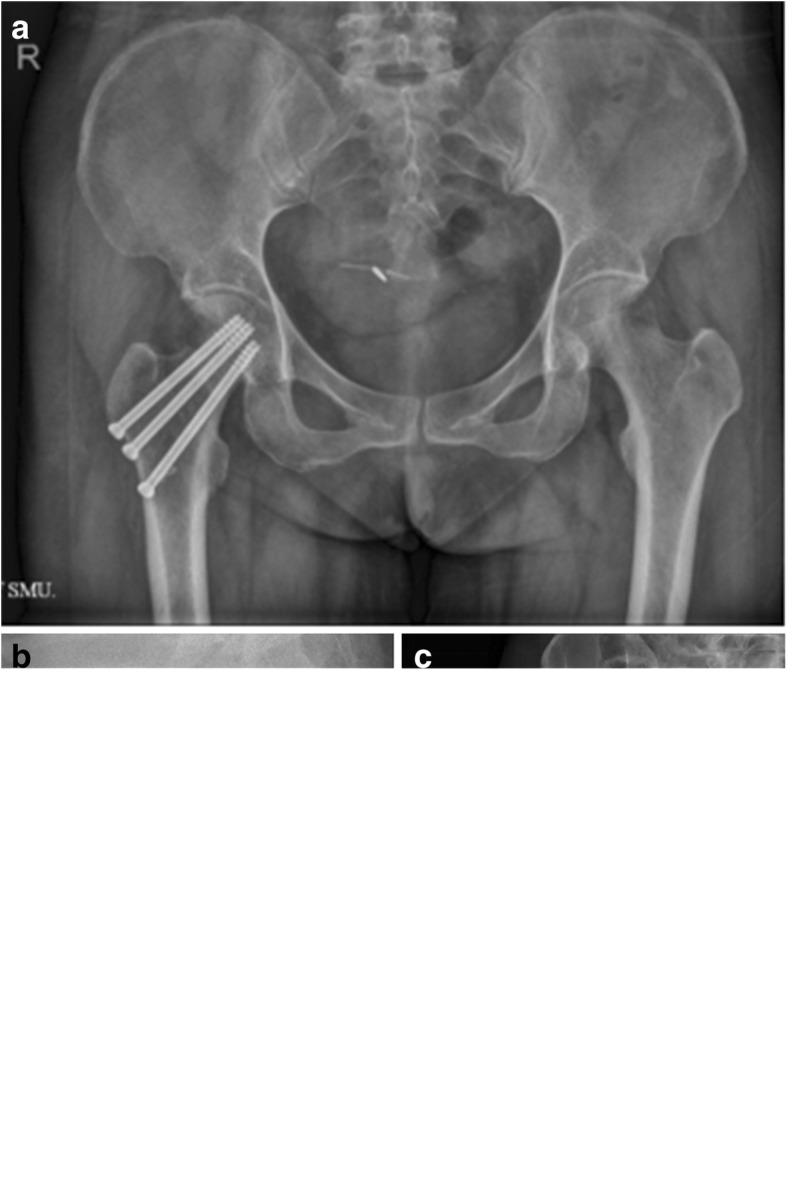
Anteroposterior view of pelvic (**a**) and lateral radiograph of the femoral neck (**b**) after closed reduction and internal fixation with three cannulated screws. Anteroposterior view of the hip after internal fixation was removed (**c**)

For patients in the MCS group, a 4–6-cm longitudinal incision was made below the greater trochanter of the femur. The iliotibial band and the vastus lateralis muscle were incised, and the lateral surface of the femur below the greater trochanter was exposed. A 3.2-mm guide wire was placed on the skin in front of the femoral neck in order to draw it close to the inferior-medial cortex of the femoral neck. Another guide wire was drilled into the femoral head from the midpoint of the lateral femoral cortical bone (parallel to the front guide wire), so that the tip of the guide wire was located behind the femoral head, and the anteversion angle was maintained within 10°. A second guide wire was then drilled in slightly above it. A third guide wire was drilled from the base of the greater trochanter, along the tensile trabecular bone, passing through the femoral neck and penetrating into the femoral head. The anteversion angle was maintained within 5° such that the guide wire was located at the more anterior part of the femoral head. The desired length of each cannulated screw was measured and the guide wire was pulled after drilling into the 7.3-mm length of the corresponding half-threaded cannulated screw. The surgeons chose to fix femoral neck fractures with screws in an inverted triangle with the depth of the cannulated screws reaching an area 3 mm under the femoral head cartilage (Fig. 4).

Regular follow-ups were performed at 6 weeks, 3 months, 6 months, and 1 year after surgery. On clinical evaluation at the latest follow-up, pain and limitation of movement were recorded. The Harris Hip Score [13] was calculated to evaluate hip function. On radiological [14, 15] or CT scan [15, 16] evaluation, the degree of union, loss of fracture alignment, and position of implant were observed. Femoral neck shortening was measured on digital radiographs using Adobe Photoshop CS 6 (Adobe Systems Inc., CA, USA), as described previously [17, 18]. The fractured hip on the most recent anteroposterior radiograph was compared with the contralateral hip on radiographs taken at the time of the injury. The uninjured side was outlined, overlapped over the fractured side, and adjusted for differences in size. Femoral neck shortening was assessed in the horizontal (abductor moment arm shortening) and vertical (femur length decrease) plane. Known diameters of screws were used to correct for differences in radiograph magnification.

### Statistical analysis

Statistical analysis was performed using SPSS version 17.0 (SPSS Inc., Chicago, IL, USA). Continuous data are expressed as mean ± standard deviation. The *t* test for two independent samples was used to analyze normally distributed data. The rank-sum test was used for data with heterogeneity of variance and non-normal distribution. Categorical data were compared using the *χ*^2^ test or the Fisher exact probability method if the theoretical frequency was less than 1. A *P* value of < 0.05 was considered statistically significant. The overall postoperative complication rate was defined as the sum of the individual rates of nonunion and fixation failure.

## Results

Table 1 shows the baseline preoperative data, including gender, age, cause of injury, fracture type, Harris Hip Scores, and time from diagnosis to surgery. Patients in the CSLP group had a mean age of 45.12 ± 5.38 years, whereas those in the MCS group had a mean age of 46.02 ± 6.23 years. The CSLP group included 55.88% of female patients and 44.12% of male patients, whereas the MCS group had 47.06% of female patients and 52.94% of male patients. The mean Harris Hip Score was 12.20 ± 2.18 in the CSLP group and 13.98 ± 2.16 in the MCS group. The mean time from diagnosis to surgery was 31.62 ± 6.79 h in the CSLP group and 32.84 ± 5.12 h in the MCS group. There were no statistically significant differences in Garden classification and OTA classification between the two groups of patients (*P* > 0.05). The causes of injuries varied between the two groups of patients. Traffic accidents were the main cause of injury (47.06%) in the CSLP group, whereas falls were the main cause of injury in the MCS group (52.94%).

**Table 1:** Preoperative clinical data

	CSLP group (%)	MCS group	*χ*^2^/*t* value	*P* value
Gender				
Male	15 (44.12)	18 (52.94)	0.54	0.51
Female	19 (55.88)	16 (47.06)		
Age (years)	45.12 ± 5.38	46.02 ± 6.23	− 0.54	0.97
Harris Hip Score	12.20 ± 2.18	13.98 ± 2.16	− 0.392	0.427
Causes of injuries				
Sport injuries	12 (35.29)	10 (29.41%)	4.76	0.03
Traffic accidents	16 (47.06%)	6 (17.65%)		
Falls	6 (17.65%)	18 (52.94%)		
Garden classification				
Type III	23 (67.65%)	21 (61.76%)	0.2576	0.6118
Type IV	11 (32.35%)	13 (38.23%)	0.2576	0.6118
OTA classification				
OTA 31-B2.3	11 (32.35)	9 (26.47)	0.2833	0.5945
OTA 31-B3.1	8 (23.53)	10 (29.41)	0.3022	0.5825
OTA 31-B3.2	9 (26.47)	8 (23.53)	0.0784	0.7794
OTA 31-B3.3	6 (17.65)	7 (20.59)	0.0951	0.7578
Time from diagnosis				
To surgery (hours)	31.62 ± 6.79	32.84 ± 5.12	− 0.72	0.528

### Postoperative results

#### CSLP group

**Fig. 5:**
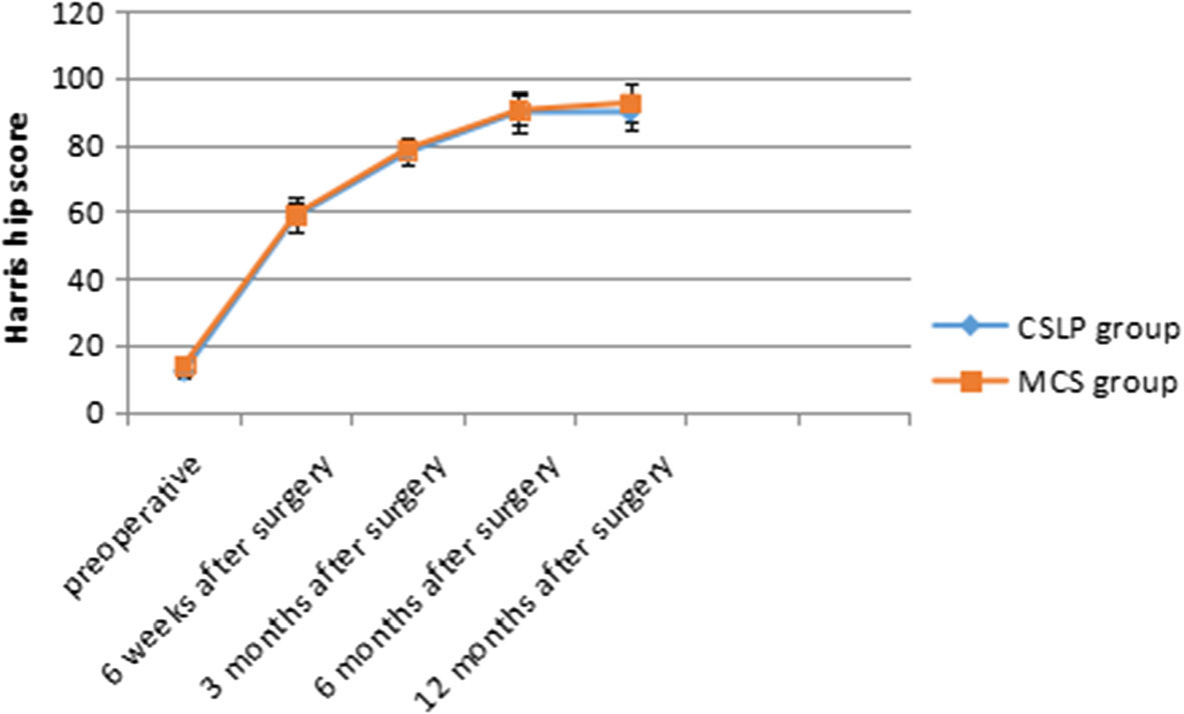
This figure illustrates the Harris Hip Scores for both groups from the preoperative to the postoperative periods. Note the increasing trend in postoperative scores for both groups

**Fig. 6:**
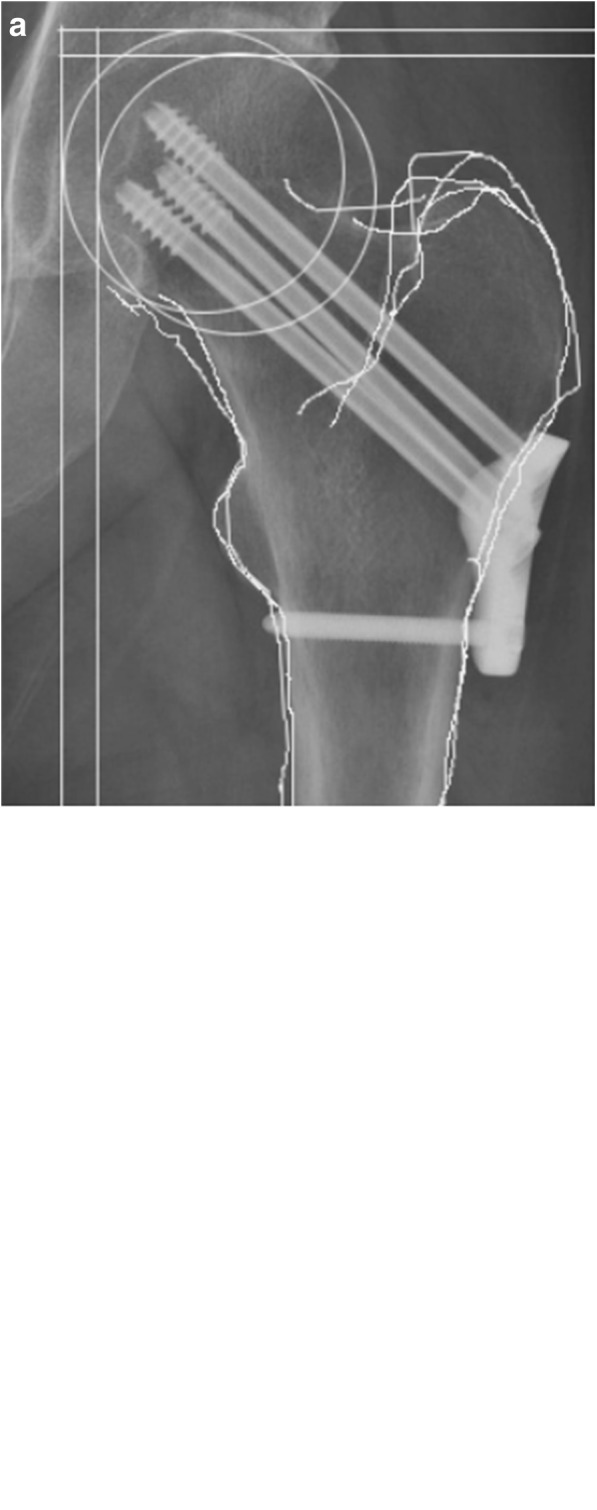
Femoral neck shortening was assessed in the horizontal and vertical plane after internal fixation with the cannulated screw locking plate (**a**) or three cannulated screws (**b**)

The mean follow-up period was 21.7 ± 4.66 months. All but two patients underwent closed reduction and internal fixation, and the mean Garden’s alignment index was 169.71 ± 4.66° on the anteroposterior position and 171.87 ± 5.46° on the lateral position. The mean Harris Hip Scores of healed patients that were revised were 59.04 ± 4.13, 78.81 ± 3.43, 90.47 ± 5.79, and 92.63 ± 5.55 at 6 weeks, 3 months, 6 months, and 1 year after surgery, respectively (Fig. 5). During the follow-up period, postoperative nonunion occurred in two patients (5.88%) and one case underwent fixation failure. Three patients (8.82%) underwent total hip arthroplasty. Mean femoral neck shortening is 5.10 mm in the vertical plane and 5.11 mm in the horizontal plane. The rates of no/mild and moderate decrease were 70.58% and 29.41% in the vertical plane and 76.47% and 23.53% in the horizontal plane. Severe decrease femoral neck shortening (10 mm or greater) did not occur in any patient both in the vertical and horizontal plane in this group (Fig. 6).

#### MCS group

The mean follow-up period was 24.8 ± 5.78 months. Two patients underwent open reduction after failure of closed reduction. The mean Garden’s alignment index was 170.59 ± 4.69° on the anteroposterior position and 171.93 ± 5.62° on the lateral position. The mean Harris Hip Scores of healed patients were 58.32 ± 5.26, 77.63 ± 3.34, 89.87 ± 4.24, and 89.74 ± 5.33 at 6 weeks, 3 months, 6 months, and 1 year after surgery, respectively (Fig. 5). During the follow-up period, postoperative nonunion occurred in eight patients (23.53%) and three patients (8.82%) developed fixation failure including screw withdrawal accompanied by displacement and angulation of the fracture site and withdrawal of cannulated screws without displacement and angulation of the fracture site. Nine patients (26.47%) underwent total hip arthroplasty in the second phase. Two patients (5.71%) received conservative treatment after removal of the internal fixation and ultimately achieved bone healing. Shortening of the femoral neck is common in this group. Mean decrease is 11.14 mm in the vertical plane and 10.51 mm in the horizontal plane. The rates of no/mild and moderate femoral neck shortening were 35.29% and 35.29% in the vertical plane and 44.12% and 32.35% in the horizontal plane. Severe femoral neck shortening occurred in 10 patients (29.41%) in the vertical plane and 8 (23.52%) patients in the horizontal plane (Fig. 6).

There were statistically significant differences in the rates of postoperative nonunion (*P* = 0.0399), shortening of the femoral neck (*P* < 0.001), and overall complications (*P* = 0.0164) between the two groups of patients during the follow-up period. There were no statistically significant differences in Harris Hip Score and fixation failure between the two groups of patients (*P* > 0.05) (Table 2).

**Table 2:** Postoperative results

	CSLP group (*n* = 34)	MCS group (*n* = 34)	*χ*^2^ value/*t* value/*z* value	*P* value
Follow-up period (month)	21.7 ± 4.66	24.8 ± 5.78	− 2.710	0.009
Garden’s alignment index				
Anteroposterior (degree)	169.71 ± 4.66	170.59 ± 4.69	− 0.253	0.801
Lateral (degree)	171.87 ± 5.46	171.93 ± 5.62	1.262	0.211
Harris Hip Score				
6 weeks after surgery	59.04 ± 4.13	58.32 ± 5.26	0.651	0.651
3 months after surgery	78.81 ± 3.43	77.63 ± 3.34	1.462	0.148
6 months after surgery	90.47 ± 5.79	89.87 ± 4.24	0.591	0.556
1 year after surgery	92.63 ± 5.55	89.74 ± 5.33	1.709	0.092
Femoral neck shortening				
In the vertical plane				
Mean decrease (mm)	5.10 ± 1.90	11.14 ± 2.78	− 11.13	< 0.0001
No/mild (0–4.9)	24 (70.58%)	12 (35.29%)	− 6.11^※^	
Moderate (5–9.9)	10 (29.41%)	12 (35.29%)		< 0.0001
Severe (10 or greater)	0	10 (29.41%)		
In the horizontal plane				
Mean decrease (mm)	5.11 ± 1.90	10.51 ± 2.78	− 10.61	0.000
No/mild (0–4.9)	26 (76.47%)	15 (44.12%)		
Moderate (5–9.9)	8 (23.53%)	11 (32.35%)	− 5.74^※^	< 0.0001
Severe (10 or greater)	0	8 (23.53%)		
Nonunion (%)	2 (5.88%)	8 (23.53%)	4.22	0.0399^★^
Failure of fixation (%)^#^	1 (2.94%)	3 (8.82%)		0.6135^★^
Overall complications (%)^&^	3 (8.82%)	11 (32.35%)	5.757	0.0164^★^

^※^Rank sum test

^★^Fisher’s exact test

^&^Overall complications = nonunion + failure of fixation

## Discussion

Although various implants have been developed for internal fixation with open or closed reduction of femoral neck fractures, predicting healing of the femoral neck by stable fixation without shortening continues to be challenging.

In our study, although the rates of fixation failure are lower in the CSLP group (2.94%) than in the MCS group (8.82%), the difference was not statistically significant. It is possible that the CSLP is a device with a fixed angle, where the placement directions and the spacing intervals of screws are fixed and cannot be adjusted. If the diameter of the femoral neck is smaller, the screws can penetrate through the bone cortex, resulting in fixation failure. However, the rates of nonunion were 5.88% in the CSLP group and 23.53% in the MCS group. Overall complications occurred in three patients (8.82%) treated with CSLP and in 11 patients (32.35%) treated with MCS. These differences were statistically significant. We assume that the major reason for the poorer results in the MCS group was insufficient mechanical stability. Because of the lack of support on the medial side behind the femoral neck and in cases where the outer cortex was unable to withstand a certain torque, multiple-screw fixation would not be ideal. Another limitation of the MCS technique is the inability to control rotation [19]. As a fixed-angle device, the CSLP consists of a small proximal femoral locking plate and four cannulated screws. The plate can provide effective support, the three screws are arranged in an inverted triangle in the femoral neck to guarantee stability, the bolt-plate locking design could help avoid loosening and withdrawal of the cannulated screws, and the other screw can be used to fix the femoral shaft, effectively preventing the rotation of the screw plate after surgery and strengthening internal fixation. The design may explain why the CSLP is superior to MCS. Biomechanical experiments have showed that, compared with MCS, a fixed-angle device can increase resistance to shear forces and reduce micromotion [11], which seems to impair the healing process. However, the CSLP itself has no longitudinal pressurization effect. If pressurization is needed initially intraoperatively, the split gaskets must be fixed onto the locking holes, and the screws are tightened so that the fracture sites are compressed. The gaskets are then removed to lock the screws and achieve the effect of compression locking. In fact, it is not clear whether dynamic compression is necessary or whether the compression achieved during fixation is sufficient. One randomized controlled trial [20] reported higher failure rates for displaced femoral neck fractures when sliding hip screws were used in a dynamic compression mode compared to static locking (33% vs. 18%).

To the best of our knowledge, there are only a few published reports on the use of a proximal femoral locking plate in the treatment of intracapsular hip fractures (Table 3). To effectively compare our treatment results with previously published results on the treatment of displaced femoral neck fractures, we used similar statistical methods for analysis. Parker et al. [21] reported a nonunion rate of 7% in 46 patients who were treated with reduction and Targon Femoral Neck Hip Screw internal fixation for displaced fractures. However, that study was conducted in an elderly cohort with a mean patient age of 75 years and had neither a control group nor randomization. Thein et al. [22] compared Targon FN and MCS in a clinical study and noted nonunion rates of 3.2% and 46.8%, respectively. Although the conclusion is consistent with ours, that study too was not a randomized controlled study but used a historical control instead. Ismail et al. performed a cross-sectional study of six displaced fractures in patients aged 17–55 years who were treated with Cloverleaf Locking Plate Fixation. The nonunion rate was reported to be 0%. Although the results were promising, only six cases of displaced fractures were analyzed. Additionally, the procedure employed was not minimally invasive, because the plate is much longer than the plate we used. Lin et al. [23] reported a nonunion rate of 7% in patients aged 21-65 years treated for displaced intracapsular hip fractures with a proximal femoral locking plate with cannulated screws, identical to the device used in our study. The nonunion rate was similar to that in our study. However, the previous study had no control group. On comparing our results with previously published results, the nonunion rate in the CSLP group in our study was lower than that in the study by Parker and higher than that in the other studies. In a recent report, Berkes et al. [24] described their experience with the Posterolateral Femoral Locking Plate, reporting a 63.6% rate of catastrophic failure in displaced fractures. They concluded that this device was not appropriate for intracapsular femoral fractures. The reason for the difference in results among the studies is not fully understood. It could be related to implant design, patient selection, follow-up time, injury-operation interval, quality of reduction, position of the internal fixation, and percentage of open reductions.

**Table 3:** A comparative literature summary of the common postoperative complications for femoral neck fractures

Author	Year	*N* of patients^☆^	Age (year)	Internal fixation	Follow-up (month)	Nonunion (%)	Fixation failure	Overall complications (%)	Control group	Study style
Lin D	2011	29	47 (21–65)	LPCS^①^	43	2 (7)	–	4 (14)	No	Prospective
Thein R	2014	31	50.9 ± 16.0	Targon FN^②^	17	1 (3.2)	–	4 (12.9)	Historical control	Prospective
Parker MJ	2009	46	75 (42–103)	Targon FN^②^	≥ 12	7 (15.2)	–	9 (19.6)	No	Prospective
Berkes MB	2012	11	71.7 ± 13.7	PLFLP^③^	23	–	7 (63.6)	8 (72.7)	Case-control	Retrospective
Ismail HD	2012	6	17–55	CLPF^④^	≥ 12	0	–	–	No	Cross-sectional study
Wang ZQ	2015	34	45.12 ± 5.38	CSLP^⑤^	21.7	2 (5.88)	1 (2.94)	3 (8.82)	Randomized controlled	Prospective

^☆^Only included those patients with displaced intracapsular hip fractures

^①^LPCS, proximal femoral locking plate with cannulated screws

^②^Targon FN, the Targon Femoral Neck Hip Screw

^③^PLFLP, the Posterolateral Femoral Locking Plate

^④^CLPF, Cloverleaf Locking Plate Fixation

^⑤^CSLP, proximal femoral cannulated screw locking plate

There is a consensus that shortening of the femoral neck is common after screw fixation of femoral neck fractures [10]. Data for femoral neck fractures treated with multiple cannulated screws indicate a shortening rate of 27 to 31% [8]. In light of this, we assess the incidence of femoral neck shortening quantitatively and qualitatively on using CSLP versus MCS in the treatment of displaced intracapsular hip fractures in young adults. In our study, the mean decrease was 5.10 mm in the vertical plane and 5.11 mm in the horizontal plane. Severe femoral neck shortening (≥ 10 mm) did not occur in any patient in either the vertical or the horizontal plane in the CSLP group. However, in the MCS group, the mean decrease was 11.14 mm in the vertical plane and 10.51 mm in the horizontal plane. Severe femoral neck shortening occurred in 10 patients (29.41%) in the vertical plane and in 8 patients (23.53%) in the horizontal plane. This implies that the CSLP also acts as a length-stable implant; locking of the screw to the side plate could prevent additional femoral neck shortening. However, MCS cannot provide enough resistance to shortening as it is based only on the friction of the bone/unthreaded portion of the screw interface [25].

We are aware of several limitations of this study. The trial was conducted at a single center and the sample size is small. Although the patients were randomized, double blinding was not performed. We did not compare time to heal between the two groups due to the limited follow-up time points. Furthermore, the 1- to 2-year follow-up is needed to visualize the shape of the femur after healing and how the constructs held up after full weight-bear in the further study.

## Conclusion

Our study demonstrated that the use of the CSLP in the treatment of displaced intracapsular hip fractures in young adults can reduce the rates of postoperative nonunion, overall complications, and femoral neck shortening. Evaluation of long-term results will require larger sample sizes and a longer follow-up period.

## Abbreviations

CSLP: Cannulated screw locking plate
DCS: Dynamic condylar screw
MCS: Multiple cancellous screws
PFLP: Proximal femoral locking plate
